# Book Notices

**Published:** 1893-11

**Authors:** 


### Book Notices.

HANDBOOK OF INSANITY, for Practitioners and Students, by Dr. Theodore Kirchhoff, Physician to the Schleswig Insane Asylum and Privatdocent at the University of Kiel. Illustrated. New York: Wm. Wood & Co. 1893, pp. 362.

The rapid appearance of handbooks of psychiatry and neurology in the English language, within the past few years, necessitates special excellence in the text-material, and decided accuracy and elegance in translation, to warrant the production in this tongue of the works of alien-speaking writers. This book of Dr. Kirchhoff's easily fulfills the first of these requirements, although its nameless translator can scarcely be held faultless; and it is probable that its popularity will be impaired by the lack of easily flowing English, and by its readily distinguishable Germanisms. Thus we cannot forgive the preservation as group-names in English of the German terms, "wahnsinn" and "verrücktheit," for certain classes of paranoia corresponding to forms of what in this country is spoken of as "monomania"; such an act only adds confusion to the already senselessly-increasing nomenclature of insanity. The endeavor to follow the German text in its intricacies and to render it in its precise equivalent in English, in many places leads to expressions not readily appreciable, and to a text more or less tiresome for continuous reading. These are the main faults of the work, and these are, of course, not to be attributed to the author.

The work is divided into two parts, a general and a special portion. In the first are considered the anatomical basis and location of normal and diseased mental processes, the nature, importance and modes of action of the various causes of insanity, the general symptoms, course, diagnosis and treatment of mental disorders, and a brief historical sketch of psychiatry. In the second, the author first outlines his mode of classification of mental disease, and then systematically discusses the subject under the following general heads: (a) *Simple mental disorders*, including melancholia, mania, periodic forms of insanity, and paranoia; (b) *Mental disorders associated with permanent anatomical changes in the brain or with general diseases*, including dementia, senile dementia, paralytic dementia, other forms of dementia with paralysis, as from cerebral syphilis, and focal diseases of the brain, epileptic and hysterical insanity, chronic and acute neurasthenic forms of insanity, toxic insanities, and feeble-mindedness.

Although following the usual endeavors to assign the brain as the site of mentality, the author clearly indicates his inclination to the reactionary belief that consciousness, and perhaps ideation, are to be regarded as functions of the general nervous system, rather than distinctly local processes. "All internal processes constitute consciousness; there are no individual modes of consciousness. The processes of consciousness are dependent on the entire nervous system, not on the cerebral cortex alone. . . . Mental diseases are not merely diseases of the brain, but diseases of the person."

In the discussion of heredity as an etiological factor, the author remarks that "children procreated during the drunkenness of otherwise healthy parents sometimes exhibit congenital mental and nervous disturbances." This may be true, but it can scarcely be held that the intoxication at time of procreation is the influencing disturbance in such a case; rather should one seek for it in a previous period of drunkenness, at the time of the development of the sperm or of the ovum. Such congenital faults rather occur in persons habitually intemperate in intoxicants; the sperm cell found under normal conditions can scarcely be held to be modified in its potentialities by the fact of its ejaculation during a temporary drunkenness of its parent body.

One must express regret that Dr. Kirchhoff has not elaborated his evidently admirable ideas upon education in the etiology of insanity, which, under the many cross-purposes of this latter-day civilization, is come to be but a synonym for mental deformity. There is an actual demand for a strong expression of dissatisfaction with our undifferentiating, mechanical methods of forcing culture, and for a clear exposition of the principles of education of the mentally unequal. "One of the purposes of an ideal education is the removal, by means of the uniform development of all the functions of body and mind, of those dangers which may lead to insanity." There is yet room for a book which shall systematically treat of the methods and measures of education of those congenitally unsound or incapable mentally.

This entire general portion of the volume is an excellently prepared disquisition upon the subjects included; a fuller consideration with reference to authorities might be asked, but as a rule, the statements of the author are reliable, and may be used by students of the subject.

The special portion of the book is for the most part clear in its statements. Perhaps the least satisfactory descriptions are to be met in the subject of paranoia and its subdivisions. In fact, it is questionable whether these subdivisions are justifiable in a volume of the modest pretensions of this. The comparative absence of illustrative cases in their course is also a feature to be regretted, nevertheless, the frequent citation of special features of individual cases may be held to compensate for this lack. The author, it must be granted, has presented the subjects of mental disorder in a philosophical and complete manner, so far as is compatible with the evident intention of the work to be a manual for the uninitiated.

The plates showing the facial types of various forms of insanity, taken with the careful description of each, constitute a valuable feature of the volume, and the mechanical execution and material of the work reflect credit upon the publishers.

A. J. S.

---

Shoemaker's Materia Medica and Therapeutics—A Practical Treatise on Materia-Medica and Therapeutics, with Especial Reference to the Clinical Application of Drugs. By John V. Shoemaker, A. M., M. D., Professor of Materia Medica, Pharmacology, Therapeutics and Clinical Medicine, and Clinical Professor of Diseases of the Skin in the Medico-Chirurgical College of Philadelphia; Physician in the Medico-Chirurgical Hospital; Member of the American Medical Association, of the Pennsylvania and Minnesota State Medical Societies, the American Academy of Medicine, the British Medical Association; Fellow of the Medical Society of London, ec.t, etc. Second edition, thoroughly revised. In two vols. The F. A. Davis Co., Publishers, Philadelphia, 1893. Cloth, pages Vol. 1, 354.

The first edition of this excellent and useful work appeared only last fall. It was highly appreciated and sold rapidly. The Journal gave a sort review of its aim and scope, and a summary of its contents, when it first appeared. We have but little to add now, except that the author has seen some defects or omissions, which no one else noticed perhaps, and hastened to correct them. The work has been thoroughly revised but we see few changes or additions, and it is issued in the same style as the first edition.

It is a most excellent work for ready reference, and is nearer complete than any of the kind we have seen; in fact, there is nothing exactly of its kind extant; for while it is a complete Materia Medica and Therapeutics, it is also a Manual of Practice of Medicine, Pharmacy and Dentistry. The "indications for and effects of drugs are given from recent bed-side observations, and Shoemaker is one of the shrewdest observers and most painstaking recorders we have. The work is up to date in every respect and will be found very handy for every day reference.

Vol. i. is devoted to Pharmacy, General Pharmacology and Therapeutics, and Remedial Agents not properly classed with drugs. No. ii, covers the balance of ground outlined above, Materia Medica and Therapeutics proper, with a valuable appendix.

We believe a student or an active physician will here find information he needs, more certainly and more promptly than in any work of reference with which we are acquainted. It is a desirable book; indispensable, in fact.

---

APPENDICITIS AND PERITYPHLITIS.—By Charles Talamon, M. D., Physician to Tenon Hospital, Paris, France. Translated by E. P. Hurd, M. D. Physician's Leisure Library. George S. Davis, Detroit, Mich. Price, paper, 25 cts; cloth, 50 cts.

This is a carefully prepared volume treating of most interesting subjects. The general practitioner will derive great benefit from reading it, and the author is to be congratulated on producing a book of so much merit.

---

INTERNATIONAL CLINICS.—A Quarterly of Clinical Lectures on Medicine, Neurology, Pediatrics, Surgery, Genito-Urinary Surgery, Gynecology, Ophthalmology, Laryngology, Otology and Dermatology, by Professors and Lecturers in the Leading Medical Colleges of the United States, Great Britain and Canada. Edited by John M. Keating, M. D., LL. D., Judson Daland, M. D., J. Mitchell Bruce, M. D., F. R. C. P., London, and David W. Finlay, M. D., F. R. C. P., Aber. Vol, I, Third Series. 1893. J. B. Lippincott Co., Philadelphia, 1893.

This volume is fully up to the high standard already established by its predecessors. The list of contributors is composed of a large number of the best men in the profession. The book being entirely clinical, the subjects are presented in the most practical, and at the same time the most interesting and instructive way possible. It really merits its great popularity and the demand for it will doubtless continue to increase.

---

AMERICAN TEXT-BOOK FOR GYNECOLOGY.—Mr. W. B. Saunders, Publisher, of Philadelphia, Pa., announces this work as ready for early issue. It is the joint work of Drs. Howard Kelley, Pryor, Byford, Baldy, Tuttle, and others, who stand before the profession for all that is progressive in gynecology. The work will contain operations not before described in any other book—notably ablation of fibroid uterus. It is designed as a profusely illustrated reference book for the practitioner, and every practical detail of treatment is precisely stated.

